# Reinforced AdaBoost Learning for Object Detection with Local Pattern Representations

**DOI:** 10.1155/2013/153465

**Published:** 2013-11-28

**Authors:** Younghyun Lee, David K. Han, Hanseok Ko

**Affiliations:** ^1^Department of Visual Information Processing, Korea University, Anam-dong, Seongbuk-gu, Seoul 136-713, Republic of Korea; ^2^Office of Naval Research, Arlington, VA 22203, USA; ^3^School of Electrical Engineering, Korea University, Engineering Buliding, Room 419, Anam-dong, Seongbuk-gu, Seoul 136-713, Republic of Korea

## Abstract

A reinforced AdaBoost learning algorithm is proposed for object detection with local pattern representations. In implementing Adaboost learning, the proposed algorithm employs an exponential criterion as a cost function and Newton's method for its optimization. In particular, we introduce an optimal selection of weak classifiers minimizing the cost function and derive the reinforced predictions based on a judicial confidence estimate to determine the classification results. The weak classifier of the proposed method produces real-valued predictions while that of the conventional Adaboost method produces integer valued predictions of +1 or −1. Hence, in the conventional learning algorithms, the entire sample weights are updated by the same rate. On the contrary, the proposed learning algorithm allows the sample weights to be updated individually depending on the confidence level of each weak classifier prediction, thereby reducing the number of weak classifier iterations for convergence. Experimental classification performance on human face and license plate images confirm that the proposed method requires smaller number of weak classifiers than the conventional learning algorithm, resulting in higher learning and faster classification rates. An object detector implemented based on the proposed learning algorithm yields better performance in field tests in terms of higher detection rate with lower false positives than that of the conventional learning algorithm.

## 1. Introduction

With advancement in image processing algorithms and proliferation of inexpensive cameras, a variety of applications such as automated CCTV based surveillance and security systems [1], human robot interface system using robot visions [2], and entertainment system using depth cameras [3] have been widely deployed in recent years. In these applications, detecting objects in image frames is a crucial step in developing practical systems. Again, a variety of topics in object detection have been explored including moving object detection [4], face detection [5] in surveillance systems, face detection for user verification [6, 7], hand detection for gesture recognition [8] in human robot interface systems, and body parts detection for motion recognition [9] in game entertainment systems.

One commonly employed method among most object detection techniques is learning based detection approach. A typical implementation of learning based object detection methods involves a pretrained detection window capable of detecting the presence of any target object within its frame as it sweeps over the entire image. In designing and training such a window, it is important that the learning algorithm engages with the most representative features of the target objects that are discernible from those of the other objects in the image. For this purpose, Haar-like features have been widely used for object detection. Haar-like feature represents a comparison of attributes such as intensity or gradient between subregions in a kernel. Since Haar-like features can be transformed into many forms depending on the location, shape, or size of the kernel, it can be useful for face detection [10–12] or license plate detection [13, 14] by choosing the best features through the learning process. However, since the method depends on the absolute values of the attributes, it may not be robust against illumination changes or motion blur.

Local pattern representation (LPR) method [15], which represents spatial relative relationships among pixels with a kernel, has recently gained spotlight among the object detection methods. Haar-like features represent differences of intensity or gradient in specific regions and may have infinite real number of feature values. In contrast, LPR represents various forms of spatial relative relationship between a specific pixel and its neighboring pixels and has a finite number of feature values. Since LPR features are based on differences rather than absolute values, it is expected that such features are robust to illumination changes and because of the finite dimensionality of the feature set, it naturally requires less memory compared to Haar-like features.

Since Ojala et al. proposed the local binary pattern (LBP) [15], a variety of LPR methods depending on the type of extracted attributes or the form of the kernel have been suggested including census transform (CT) [16], modified census transform (MCT) [17], local gradient patterns (LGP) [18], and local structure patterns (LSP) with cross-shaped kernel [19]. For design of LPR based classifiers, techniques such as template matching [20], support vector machine [21], linear programming [22], or AdaBoost learning have been used. AdaBoost algorithm is a well-known classifier combination method to construct a strong classifier with weak classifiers [23, 24]. Due to its effective generalization capability coupled with low implementation complexity, Adaboost method with LPR has become one of the most popular and effective classification tools in face alignment [5], frontal face classification [25], license plate detection [19], and so on.

In this paper, a reinforced Adaboost learning algorithm using LPR features is proposed. In particular, we introduce an optimal selection of weak classifiers minimizing the cost function and derive the reinforced predictions based on a judicial confidence estimate to determine the classification results. For the decision of classifications, the weak classifier of an original Adaboost used in [5, 17–19, 25] produces an integer valued prediction of either +1 or −1. However, the weak classifier of the proposed method produces a real value which reflects the confidence level of the prediction. This enables us to update the sample weights individually depending on the confidence level of prediction of the weak classifier, unlike the conventional learning algorithm wherein the entire sample weights are updated at the same rate. Consequently, the proposed learning algorithm is compact with a smaller number of weak classifiers compared to the conventional learning algorithms but is capable of producing a strong classifier with the same performance. As a result, a faster and more accurate detector can be constructed for object detection.

Most classification methods proceed by minimizing the training error. Our approach in Adaboost algorithm is to minimize the upper bound of training error. For this purpose, we employ an exponential criterion and Newton's method in optimization. We use the exponential criterion as a cost function for its differentiability to set the upper bound on the misclassification rate for optimizing the model of classifiers. Since the exponential criterion is monotonic and smooth, the classifier model can be optimized by Newton's optimization method without the risk of falling into a local minimum. When implementing an LPR based weak classifier, a lookup table is employed in order to produce the classification results by finding the designated predictions corresponding to the LPR feature values.

The rest of the paper is organized as follows. The concept of LPR and design of the classifiers are described in Section 2.  Section 3 explains the proposed learning algorithm in detail. Experimental results are provided in Section 4. Finally, conclusions are drawn in Section 5.

## 2. Local Pattern Representations and Design of Classifier

Selection of feature sets is obviously very important for detecting the intended objects from background, but it is particularly crucial in learning based classifiers. LPR is a well-known feature extraction method that represents spatial relative relationship between a specific pixel point and its neighboring pixels. MCT may be an example, which is defined as an order set of binary comparison of pixel intensities between neighboring pixels in the kernel and their mean intensity. MCT at a specific point  **x**  is defined as

(1) $$\begin{array}{c} \Gamma \left(x\right)=\underset{y\in N^{\prime }\left(x\right)}{⨂}\zeta \left(\overset{-}{I}\left(x\right),I\left(y\right)\right), \end{array}$$

where *N*′(**x**) is a local spatial neighborhood in kernel, **I**(**x**) is the intensity at pixel **x**, and $\overset{-}{I}\left(x\right)$ is the mean intensity in this neighborhood. *ζ*(·) is the comparison function which is equal to 1 if $\overset{-}{I}\left(x\right)<I(y)$ and is equal to 0 otherwise. The concatenation operation which converts a binary string into a decimal number is denoted by ⊗.

**Figure 1:**
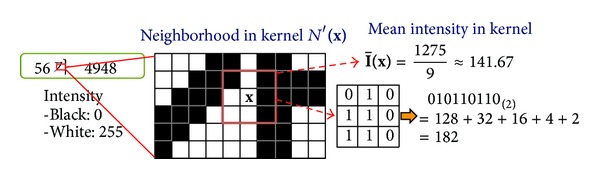
An example of the feature extraction of an image region in a vehicle license plate using MCT.

Figure 1 shows an example of MCT feature extraction with a 3 × 3square-shaped kernel from a license plate image. In the first step, the mean intensity of nine neighborhood pixels in the kernel including itself is calculated at a specific pixel location. Subsequently, in the second step, the result of the first step is compared to the mean intensity and the intensity of each location in the kernel. In the third step, the intensity values are converted into a binary string whose values are equal to 1 if the intensity of the pixel is greater than the mean intensity and is equal to 0 otherwise. In the final step, the MCT feature value is obtained by converting the binary string into a decimal number. After the conversion, the MCT with a 3 × 3square-shaped kernel can have integer feature values ranging from 0 to 510. Between MCT and other LPRs such as LBP, LGP, and LSP, their differences depend only on the type of kernel used and the attributes compared. All their feature values are in integers.

Typical feature extraction methods widely used in pattern recognition produce continuous real-valued features such as differences of intensity, magnitudes of edge, or directions of edge. For example, when the magnitude of an edge grows, the feature value becomes larger, while when the magnitude of an edge shrinks, the feature value becomes smaller. Therefore, a classifier with these types of features can be implemented simply by selecting a threshold boundary in terms of metric distance. However, the LPR has discrete integer-valued attributes as feature values. Each integer value of features represents its own independent pattern and the feature value does not have metric distance characteristic [26]. Therefore, in implementation of LPR based classifiers, a metric distance based threshold boundary cannot be used as a basis for decision. Instead of determining the threshold with a set of real numbers, these classifiers typically employ a lookup table as means of determining the classification boundaries.

**Figure 2:**
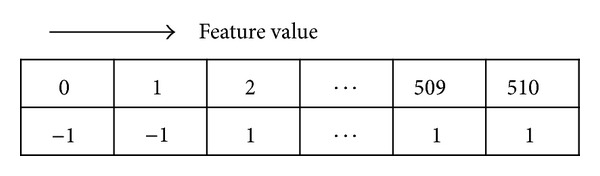
Example of classifier using lookup table.

An example of a classifier using a lookup table can be implemented as shown in Figure 2. The number of the first row represents the LPR feature values and the second row indicates the corresponding prediction of the classifier. In short, the classifier produces the decision results by finding the designated prediction corresponding to each feature value.

**Figure 3:**
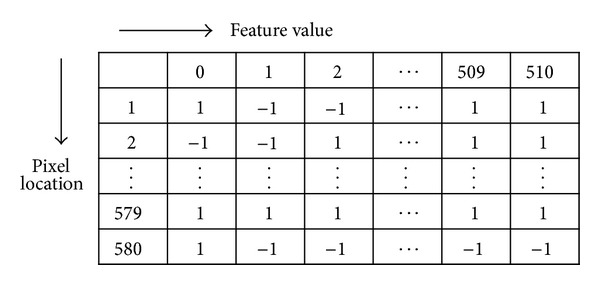
Example of weak classifiers using lookup table.

The LPR based method uses not only finite-integer numbers as feature values but also a small number of pixel locations within an image patch as candidates of weak classifiers.  Figure 3 shows an example of weak classifiers using a lookup table used in actual object detection. The numbers of the first row and the first column represent the LPR feature values and the candidates of the weak classifier, respectively. The Adaboost learning with LPR selects the best pixel location having minimum error rate. So, each pixel location can be a candidate of a weak classifier. The key advantage of using a lookup table is fast computation since the number of combining operations for evaluating a strong classifier never exceeds the number of pixel locations in an image patch even though the number of iterations for selecting weak classifiers may become large. A detailed explanation related to this is described in Section 3.

## 3. Learning AdaBoost Based Classifier

### 3.1. Selection of Weak Classifiers

In the AdaBoost algorithm, the function of the strong classifier *H*(*x*) is updated as *H*(*x*) = *H*(*x*) + *h*_*t*_(*x*) at each step *t* with the function *h*_*t*_(*x*) chosen to minimize a cost function. Here, we adopt the most popularly used exponential criterion as the cost function,

(2) $$\begin{array}{c} J=E\left[\exp\left(-yH\left(x\right)\right)\right], \end{array}$$

where *E*[·] represents expectation and *y* is 1 or −1 as the desired label of *x* [27, 28]. Now, we have to find a way to optimize the exponential criterion for Adaboost learning. In this paper, we use the Newton's method for optimizing the exponential criterion [28] since the characteristic of an exponential criterion is monotonic and smooth. The classifier model, in this case, can be optimized by Newton's method without the risk of falling into a local minimum. The Newton's optimization of the function of exponential criterion *J*(*H*(*x*) + *h*(*x*)) = *E*[exp⁡⁡(−*y*(*H*(*x*) + *h*(*x*)))] with respect to *h*(*x*) at each step can be described as in the following equation:

(3) $$\begin{array}{c} H\left(x\right)⟵H\left(x\right)-\left(\frac{\partial J\left(H\left(x\right)+h\left(x\right)\right)/\partial h\left(x\right)}{\partial^{2}J\left(H\left(x\right)+h\left(x\right)\right)/\left(\partial h{\left(x\right)}^{2}\right)}\right). \end{array}$$

By computing the first and second derivatives of *J*(*H*(*x*) + *h*(*x*)) at *h*(*x*) = 0, (4) is obtained as

(4) $$\begin{array}{c} H\left(x\right)⟵H\left(x\right)+\left(\frac{E\left[ye^{-yH(x)}\;|\;x\right]}{E\left[e^{-yH(x)}\;|\;x\right]}\right)\text{.} \end{array}$$

A weighted conditional expectation *E*_*w*_(·∣*x*) is defined as

(5) $$\begin{array}{c} E_{w}\left[g\left(x,y\right)\;|\;x\right]\overset{\mathrm{def}}{=}\frac{E\left[w\left(x,y\right)g\left(x,y\right)\;|\;x\right]}{E\left[w\left(x,y\right)\;|\;x\right]}, \end{array}$$

where *w*(*x*, *y*) = exp⁡⁡(−*yH*(*x*)) are the weights for the training examples. Finally, we can get the updated form as shown in (6) from (4) by incorporating (5) as

(6) $$\begin{array}{c} H\left(x\right)⟵H\left(x\right)+E_{w}\left[y\;|\;x\right]. \end{array}$$

As shown in (6), we can see that the weak classifier *h*_*t*_(*x*) can be chosen in the form of *E*_*w*_[*y* | *x*] which essentially represents the best estimate of the decision based on the observation *x*. In the next section, we discuss our two main contributions in this paper. That is, how the best weak classifier for constructing a strong classifier is selected and how we derive a reinforced prediction of *h*_*t*_(*x*) in the learning procedure of LRP feature based classifiers implemented by lookup tables.

### 3.2. Learning of an LPR Based Classifier

Algorithm 1 shows the proposed Adaboost learning procedure of the LPR based classifiers. Prior to starting with the learning process, training samples for learning of classifiers are prepared. Positive samples consist of the patch images of target objects, and negative samples consist of the patch images from the background containing nontarget objects. In the first step of the learning procedure, the weights for positive samples and negative samples are initialized. In the second step, the best weak classifier is selected for each round. In the third step, a pixel classifier of a single pixel location is generated by constructing a weak classifier at pixel location **x** selected in the second step. Finally, a strong classifier is made by the sum of all the pixel classifiers.

**Figure 4:**
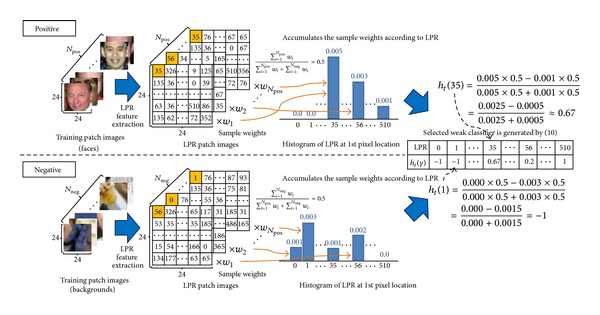
Selection of weak classifier for each round in AdaBoost learning. At first, LPR images are extracted from the training images. The histogram of LPR is generated by accumulative weights of samples according to the LPR feature values of the same pixel location. Assuming that the 1st pixel location is the best pixel location with the smallest error rate, it can be selected as a weak classifier for that round in AdaBoost learning. The selected weak classifier is described as a lookup table which is generated by (8), and the real values in the lookup table represent the confidence level of prediction.

A detailed description of the steps to select the weak classifiers as listed in Algorithm 1 is given below. First, histograms of LPR from positive samples and negative samples are generated as shown in Algorithm 1  (2)(a). A histogram is generated by taking an accumulative weight of samples, according to the LPR feature values of the same pixel location. In this respect, a specific LPR feature value occupies one of the bins of the histogram. For example, in Figure 4, consider that there are *N*_pos_ positive samples and *N*_neg_ negative samples with the number of pixels in a sample to be 24 × 24 = 576 while LPR feature values range from 0 to 510. The histogram of all 576 pixel locations accumulates the sample weights that fall into each of the bins from positive samples and negative samples. Therefore, this would produce 576 histograms for both positive samples and negative samples with each histogram having 511 bins. In order to design a weak classifier for the 1st pixel location, the histograms of both the positive samples and the negative samples are compared by searching for larger accumulated weights bin by bin. The decision of classification for one bin is made depending on the side having a larger accumulated weight. Then, an error rate of bin classification will be the value of the accumulated weight of the side having the smaller weight. For example, if the accumulated weights of the 35th bin at the 1st pixel location are 0.005 and 0.001 from positive samples and negative samples, respectively, the decision of classification for the LPR feature value at the pixel location is to be positive and the error rate of classification for the LPR feature value at the pixel location is 0.001. The total error rate of a certain pixel location is calculated by adding its bin error rates as shown in Algorithm 1  (2)(b).

The best pixel location with the smallest error rate is chosen as the weak classifier for each round in AdaBoost learning. However, if the number of pixel locations for combining a strong classifier is limited to less than *n* and the *n* pixel locations are already selected in the previous rounds, the best pixel location must be chosen by comparing the error rates among the selected pixel locations in previous rounds. By doing so, no matter how the number of rounds increases, the number of combined classifiers for constructing a strong classifier can be fixed, while the performance of the strong classifier improves.

With *E*_*w*_[*y* | *x*] considered as the best selected weak classifier, the lookup table for the weak classifier can be formulated as follows. Since *y* is a binary label of either +1 or −1, *E*_*w*_[*y* | *x*] can be expressed as

**Figure 5:**
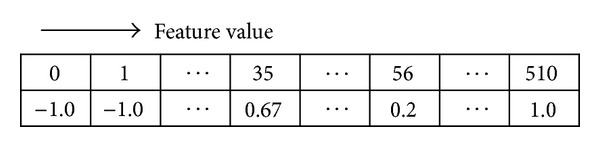
Example of weak classifier in the proposed method.

(7) Ew[y ∣ x]=Pw(y=1 ∣ x)−Pw(y=−1 ∣ x)=Pw(x ∣ y=1)P(y=1)Pw(x)−Pw(x ∣ y=−1)P(y=−1)Pw(x)=Pw(x ∣ y=1)P(y=1)−Pw(x ∣ y=−1)P(y=−1)Pw(x ∣ y=1)P(y=1)+Pw(x ∣ y=−1)P(y=−1),

where *x* is an input vector, *y* is a desired label, and *P*_*w*_(*x* | *y*) is a probability of *x* given *y*. We define that *g*(**x**, *γ*) is the value of the *γ*th bin of the histogram at pixel location **x**, and *P*_pos_ and *P*_neg_ are the ratios of the sum of positive weights and negative weights, respectively, to the sum of all the weights, *P*_pos_ = ∑_*j*∈pos_ *w*_*j*_^*t*^/∑_*i*_ *w*_*i*_^*t*^ and *P*_neg_ = ∑_*j*∈neg_ *w*_*j*_^*t*^/∑_*i*_ *w*_*i*_^*t*^. In (7), *P*_*w*_(*x* | *y* = 1) and *P*_*w*_(*x* | *y* = −1) can be replaced with *g* ^LP^(**x**, *γ*) and *g* ^BG^(**x**, *γ*), respectively. The probability that *y* is equal to 1 or −1 can be replaced with *P*_pos_ or *P*_neg_, respectively. Hence, the selected weak classifier is described as a lookup table which is generated by the following equation:

(8) $$\begin{array}{c} h_{t}\left(\gamma \right)=\frac{g_{t}^{\text{pos}}\left(x_{t},\gamma \right)\cdot P_{\text{pos}}-g_{t}^{\text{neg}}\left(x_{t},\gamma \right)\cdot P_{\text{neg}}}{g_{t}^{\text{pos}}\left(x_{t},\gamma \right)\cdot P_{\text{pos}}+g_{t}^{\text{neg}}\left(x_{t},\gamma \right)\cdot P_{\text{neg}}}, \end{array}$$

where **x**_*t*_ is the selected pixel location at round *t* and *γ* is the LPR feature value. The classifier using the lookup table generated from (8) which differs from the classifier of Figure 2 is presented in Figure 5. The values in the lookup table shown in Figure 2, being for a binary decision, are either 1 or −1, while the values in the lookup table shown in Figure 5 are real numbers representing the confidence level of reinforced prediction. If the confidence level of prediction is a positive number, the result of classification is positive; otherwise, it will be negative. A large absolute number implies that the prediction is highly confident.

The real numbers obtained in this sense can be considered as “reinforcement” in the AdaBoost learning for the following reasons. The “reinforcement learning” is a term usually coined to a state transition process that describes a learning action taking place so as to maximize the cumulative reward. In the case of AdaBoost learning, the constructed classifier is guided with reward in the direction of the fastest route toward the final convergence [29]. In particular, the classifier requires a cumulative reward feedback on the result of the classification. In this respect, the real-valued prediction based on some measures of confidence level can be used an effective feedback for fast convergence. In the AdaBoost learning, the constructed strong classifier can be optimized by updating the weights of the samples that indicate their importance for the classification. On each round, the weight of the correctly classified samples is decreased, whereas the weight of the misclassified samples is increased. Subsequently, the new classifier of the next round can focus on the samples which have avoided correct classification thus far.

In contrast, the conventional learning method based on an original AdaBoost used in [5, 17–19, 25] produces a binary prediction of either +1 or −1. In this case, the entire sample weights are updated by the same rate regardless of the confidence levels of predictions. Consequently, it may lead to slow convergence requiring many rounds of iterations for constructing weak classifiers. However, in our reinforced AdaBoost learning method, the weak classifier *h*_*t*_ produces a real-valued prediction which reflects the confidence level of the prediction. Hence, the amount of change of the updated weight of each sample depends on the confidence level of prediction. In this case, the weight of the correctly classified sample with high confidence is significantly reduced to be ignored in the next round, whereas the weight of the misclassified sample with high confidence is significantly increased to be concentrated in the next round. By doing so, the number of the selection of weak classifiers can be reduced. Therefore, the proposed update method can be called “reinforcement learning” that can guide the classifier toward the fastest route toward convergence.

The update of the additive model to the weight of the *i*th sample, *w*_*i*_(*x*, *y*) = exp⁡⁡(−*yH*(*x*)), is applied by

(9) wit+1(x,y)=exp⁡[−y{H(x)+h(x)}]=exp⁡(−yH(x))exp⁡(−yh(x))=wit(x,y)exp⁡(−yh(x)).

By including a normalization step, the update for weight of the ith sample at round *t* is obtained by the following equation:

(10) $$\begin{array}{c} w_{i}^{t+1}=\frac{w_{i}^{t}\exp\left[-y_{i}h_{t}\left(\Gamma_{i}\left(x\right)\right)\right]}{Z_{t}}, \end{array}$$

where *Z*_*t*_ = ∑_*i*=1_^*N*^ *w*_*i*_^*t*^exp⁡⁡[−*y*_*i*_ *h*_*t*_(Γ_*i*_(**x**))] is the normalization factor, Γ_*i*_(**x**) is the LPR feature value at pixel location **x** of the ith sample, respectively.

After selecting *T* weak classifiers, a pixel classifier of a single pixel location is constructed as

(11) $$\begin{array}{c} p_{x}\left(\gamma \right)=\sum_{t=1}^{T}h_{t}\left(\gamma \right)I\left(x=x_{t}\right), \end{array}$$

where *I*(·) is an indicator function that takes 1 if the argument is true and 0 otherwise.

Finally, it is possible to construct a final strong classifier combining pixel classifiers. The final strong classifier determines a test sample into positive samples for positive values and negative samples for negative values as

(12) $$\begin{array}{c} H\left(\Gamma \right)=\mathrm{sign}\left(\sum_{x}p_{x}\left(\Gamma \left(x\right)\right)\right). \end{array}$$

As affirmed previously in Section 2, the advantage of LPR based method we propose here is its fast computation due to a small number of candidates of weak classifiers. For example, the Haar-like feature based method selects a large number of weak classifiers from a significantly larger set of candidates of weak classifiers to construct a strong classifier. The final strong classifier can be described as

(13) $$\begin{array}{c} H\left(x\right)=h_{13}^{1}\left(x\right)+h_{7}^{2}\left(x\right)+h_{108}^{3}\left(x\right)+\cdots +h_{83}^{T}\left(x\right), \end{array}$$

where a superscript means the sequence number of the selected weak classifier and a subscript refers to the index of candidates. This method requires the selection of a large number of weak classifiers, *T*, from even significantly larger pool of candidates. Since each of the selection processes is mostly independent, the method requires a very large number of computational operations.

For comparison, consider that the proposed method using LPR and lookup table needs to select the same number of weak classifier *T* among *n* candidates. For a typical LPR implementation, *T* and *n* may be of values that are approximately a few thousands and a few hundreds, respectively. Thus, due to the repeated selection process where *T* is greatly larger than *n*, it is obvious that some weak classifiers will be selected many times. To generate a pixel classifier *p*(*x*), the proposed method calculates the combination operation of the weak classifiers in advance at the repetitively selected pixels. Hence, the final strong classifier of the proposed method can be derived as

(14) H(x)=h11(x)+h22(x)+h13(x)+⋯+h6T(x)=∑i∈G1h1i(x)+∑i∈G2h2i(x)+⋯+∑i∈Gnhni(x)=p1(x)+p2(x)+⋯+pn(x),

where *n* is the number of the candidates of weak classifier and *G*_*j*_ is the group of the sequence numbers of selected weak classifiers having index *j*. In short, the combination of the weak classifiers that have been selected during the training process with the same index may be calculated in advance. Hence, only *O*(*n*) computational operation is required to obtain *H*(*x*) in (14), which is less than the computation load *O*(*T*) of *H*(*x*) in (13), where *n* ≪ *T*.

In summary, we have selected the best weak classifier with the smallest error rate for constructing a strong classifier and derived a reinforced prediction of weak classifier in the Adaboost learning. Our strategy is to

1. generate the histograms of LPR from positive samples and negative samples,
2. select the best pixel location from comparing histograms with the smallest error rate as the weak classifier,
3. describe the selected weak classifier as a lookup table with the reinforced predictions which is derived from *E*_*w*_[*y* | *x*], and
4. update the sample weights individually depending on the confidence level of reinforced prediction.

In the next section, we will show the effectiveness of our proposed reinforced learning method through two performance evaluation criteria: accuracy and learning speed.

## 4. Experimental Results

This section describes how we evaluated the performance of the proposed learning algorithm. In order to validate the effectiveness of the learning algorithm proposed in this paper, the proposed learning algorithm is compared in terms of accuracy and learning speed with a conventional learning algorithm using the same features [17–19, 25]. We employ MCT [5, 17, 19, 25] as feature because of its superior performance over illumination changes compared to that of LBP and CT. In these experiments, we use MCT with a  3 × 3  square-shaped kernel as the feature sets.

### 4.1. Database

For training of the classifier and performance assessment of our proposed algorithm, we used a dataset which consists of human face images and vehicle license plate images. To obtain the face training images, we took the following steps. First, 4391 images that contain more than one face were collected from the Web, and then, four points on the coordinates of the rectangle framing the face were collected for each face in the images. All training face images were scaled and aligned to 24 × 24 pixels. From each selected frame, additional training frames were generated by randomly translating the frame within 8 pixels and rotating within 10 degrees to a total of 100,000.

**Figure 6:**
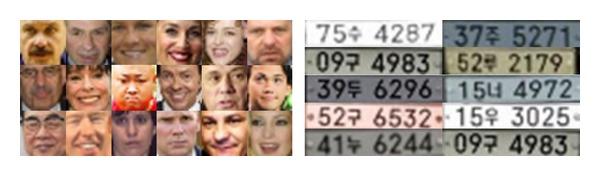
Training image examples of faces and license plates.

For the license plate training images, a digital video camera captured images from a static location on a street and a parking lot. A total of 10,000 still images that contained more than one license plate were selected from video. All license plates were cropped manually and scaled and aligned to 12 × 60 pixel frame. As in the case with facial images, a total of 100,000 training license plate images were generated by randomly translating and rotating each selected frame within 5 pixels and 10 degrees, respectively. Some examples of face and license plates are shown in Figure 6. The background images were collected by randomly selecting 150,000 frames from an arbitrary image set that does not contain any face or any license plate. The size of a background image is the same as the face images or the license plate images. Since object (face or license plate) detection test needs far more background images as negative samples, additional images were generated by the same method.

### 4.2. Cross-Validation Test

To evaluate the accuracy performance of the classifier, a 5-fold cross-validation test in terms of error rate was performed with 100,000 positive samples (faces, license plates) and 150,000 negative samples (backgrounds). All samples were divided into five groups that have 20,000 positive samples and 30,000 negative samples each. A single group was retained as the validation set for testing the trained classifier, and the remaining four groups were used as the training set. Thus, the cross validation process can be repeated five times with each validation set. The five results of error rates can be averaged to produce the overall performance.

**Figure 7:**
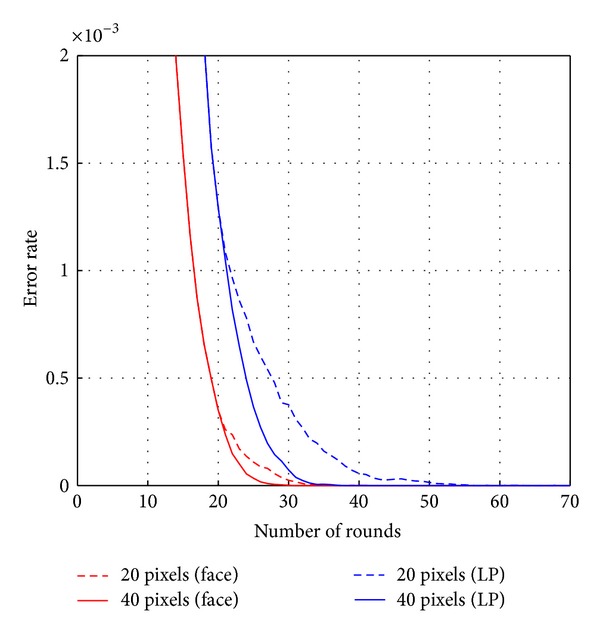
Training error according to the number of pixel locations.

**Figure 8:**
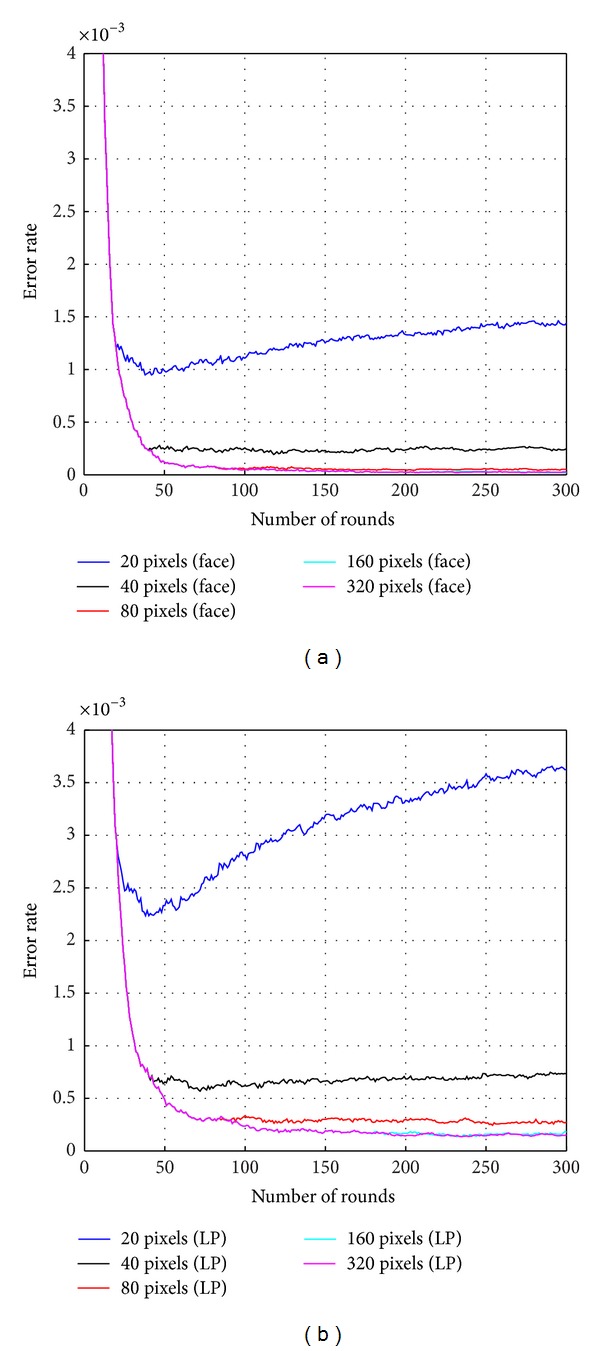
(a) Testing error according to the number of pixel locations on face DB. (b) Testing error according to the number of pixel locations on license plate (LP) DB.

Figures 7 and 8 show the comparison of the performances according to the number of pixel locations combined and used in the strong classifier. The numbers of pixels that were compared were 20, 40, 80, 160, and 320. Horizontal axis marks the number of rounds for Adaboost training while vertical axis shows the error rate. The total number of rounds for Adaboost training was 1000.  Figure 7 shows the results of training error. Red lines represent the results on face DB, and blue lines represent the results on license plate DB. Dotted lines and solid lines represent the results when the numbers of combined pixels are 20 and 40, respectively. As shown in Figure 7, the training error rate for the face DB converges to zero with 20 pixels at round 37 and 40 pixels at round 31. The training error rate for the license plate DB converges to zero with 20 pixels at round 57 and 40 pixels at round 38. The training error rate of 80, 160, or 320 is the same as the training error rate of 40. Thus, the training error rate converges to zero rapidly when the number of combined pixels becomes larger.  Figure 8 shows the results on testing error for face DB and license plate DB, respectively. Through the experimental results, the testing error rate is closed to zero when the number of combined pixels becomes larger at the same round. For the number of pixels above 40, it is observed that the testing error rate results diverged, although the results of training error rate were uniform. It is noted that when the number of combined pixels is too low to construct the strong classifier, such as for 20 pixels, the testing error rate decreases up to round 40 but increases beyond round 40. It is evident for the case of small number of pixels that overfitting occurs in larger number of rounds. However, it seems that overfitting does not occur when the number of combined pixels becomes greater than 80.

Table 1 shows the execution time of classification and the number of misclassified samples for 200,000 test samples on a MATLAB platform. The classifier used to test was trained with 20,000 positive samples and 30,000 negative samples that were not included in the test samples. For the assessment to be reliable, the experiments for computing execution times were repeated ten times and the total execution time and their averages were calculated. Each column of the table represents the number of combined pixels used for the strong classifier and all the tested classifiers were trained through the same 1000 rounds. When the number of pixels decreases, the processing speed becomes faster while the error rate increases. On the contrary, when the number of pixels increases, the processing speed becomes slower and the error rate decreases. Therefore, in order to configure a cascade classifier as shown in [17–19], the strong classifier consisting of a small number of pixels was placed at earlier stage and a large number of pixels at later stage.

**Figure 9:**
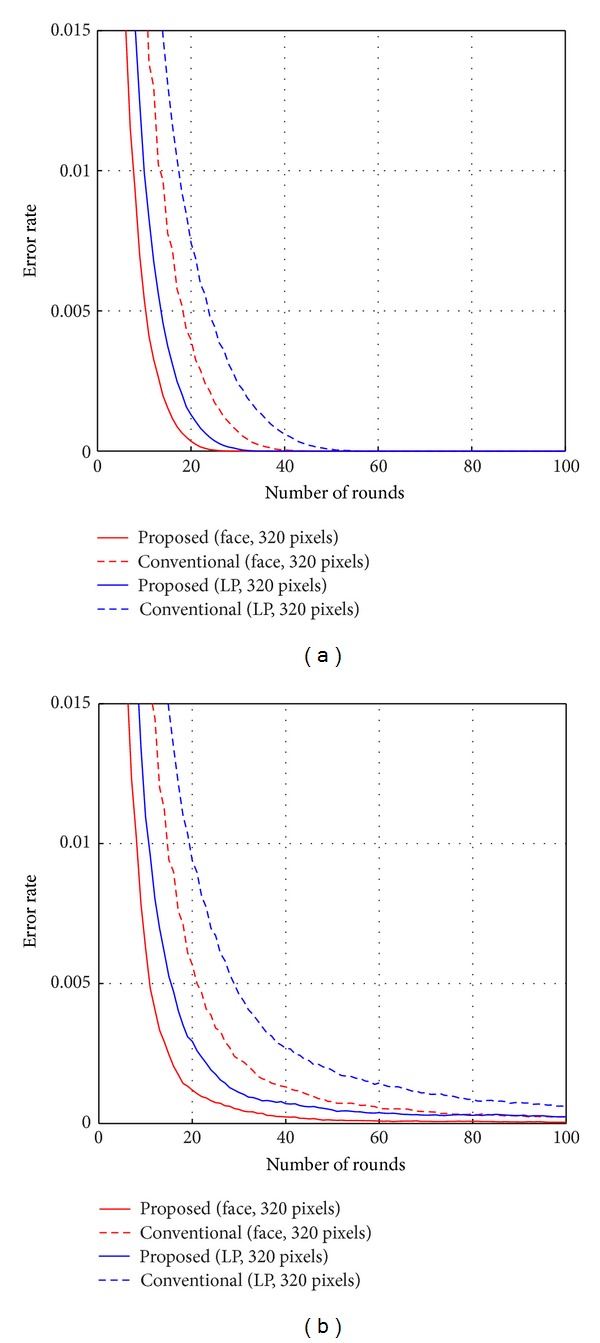
(a) Comparison of learning methods for training error. (b) Comparison of learning methods for testing error.

Figure 9 shows a comparison of learning algorithms composed of the proposed algorithm and the conventional algorithm used in [17–19, 25]. The number of combined pixels for the strong classifier was 320. Red lines and blue lines represent the results on face DB and license plate DB, respectively. Dotted lines illustrate the results of the conventional algorithm, and solid lines depict the results of the proposed algorithm. For the training error rate as shown in Figure 9(a), the proposed algorithm has better performance since its training error rate converges to zero quicker with both databases. According to the results, the training error rate for the face DB converges to zero with the proposed algorithm at round 31 and the conventional algorithm at round 48. The training error rate for the license plate DB converges to zero with the proposed algorithm at round 38 and the conventional algorithm at round 61.  Figure 9(b) shows a comparison of performance of the proposed and conventional algorithms for testing error. Through the experimental results, it can be claimed that the error rate of the proposed algorithm converged to zero faster than that of the conventional algorithm. Therefore, the classifier based on the proposed algorithm exhibited better performance with the same number of rounds compared to the classifier based on the conventional algorithm. Subsequently, the training time can be shortened over the conventional method as it is clearly shown for a field test described in the following section.

### 4.3. Field Test

**Figure 10:**
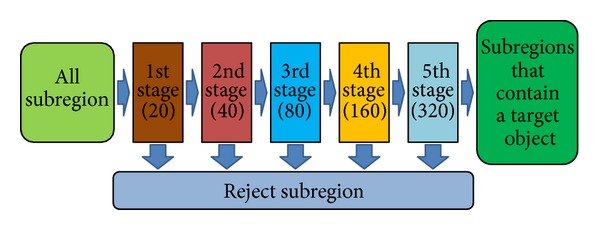
Cascade structure of detector.

To evaluate the object detection performance based on the proposed learning algorithm, tests were conducted to detect the location of target objects in still images. The procedure of detection is described as follows. A detecting window having the same size as its training image can determine whether an image within a window contains a target object or not. Hence, the detecting window slides across the image in raster scan format and classifies each subregion. Since the size of the detecting window is fixed as that of its training image, the detecting window should progressively scan like in pyramid formation, which is a collection of downsampled images, in order to detect target objects of various sizes. The detector consists of a cascade structure of five stages, and the maximum number of pixel locations for each stage is 20, 40, 80, 160, and 320, respectively. Test images are classified sequentially from stage 1 to stage 5. The images classified as a target object are carried over to the next stage, and only the images classified as target objects at all stages can be confirmed as the target object.  Figure 10 shows the cascade structure of the detector. The number in parentheses refers to the maximum number of combined pixel locations. At the earlier stage of the detector most of the search area is examined coarsely but quickly using the selected small number of combined pixel classifiers. Then, at the later stage of the detector a smaller search area is slowly but accurately examined with the selected larger number of combined pixel classifiers. The target objects of these tests were defined as faces or license plates. The positive training sets for each stage consisted of 100,000 face images and 130,000 license plate images, respectively. The negative training sets were composed of 150,000 background images for the face detector and 210,000 background images for the license plate detector. The positive training sets were equally applied to all stages. At each stage, those negative training sets that were classified as “background” were discarded in the following stage and replaced by the other background images that have not been used in previous stages.

After preparing the training sets, the MCT features were extracted from all the training sets and the detectors were trained according to the proposed learning algorithm. The number of rounds is progressively increased until the training error becomes zero or it reaches the maximum number of rounds 1500. For comparison of the performance of the proposed algorithm against the conventional method, the detector based on the conventional algorithm in [17–19, 25] was implemented and compared by applying all test conditions of the proposed algorithm stated previously.

Tables 2 and 3 summarize the trained classifiers for face detection and license plate detection. The numbers in rows of “Rounds” and “Pixels” mean the required number of rounds for the training error to become zero and the final number of combined pixels in the classifier, respectively. The number in the row of “Dataset” means the number of datasets for training of the classifier at each stage. The number in parentheses refers to the number of actual datasets that was used to fill the number of negative training datasets that were misclassified in the previous stages. In stage 4 and stage 5, it became difficult to fill the required number of negative samples because most of the negative samples were removed in the previous stages. Therefore, only a small number of negative samples from the set originally numbered in billions were available for the training. As an example, in stage 5 of the face detector, only 0.32 × 10^5^  negative samples were used for training among the 3.76 × 10^9^ negative samples.

As alluded in Section 4.2, the proposed learning algorithm requires smaller number of rounds than the conventional learning algorithm for the training error to become zero. Also, in some cases of license plate detection, a number of pixel locations smaller than the maximum possible were selected at stages 4 and 5, resulting in the detection of speed improvement. The value of “Dataset” in parentheses for the proposed learning algorithm was larger than the conventional learning algorithm, meaning that the classifier based on the proposed learning algorithm at earlier stages can remove more negative samples. Therefore, it can be said that these results indicate that the proposed learning algorithm is more efficient compared to the conventional learning algorithm.

The face detector has been tested on two commonly used databases. One is the CMU + MIT database [30] which has 107 images with 450 visible frontal faces excluding hand-drawn images, cartoons, and images containing small faces. The other is the BioID database [31] which has 1210 images with 1210 frontal images excluding images containing faces much too large for the object detector. The license plate detector has been tested on 248 real field images that contain 287 visible license plates under various weather conditions. The scale parameter and shifting parameter for both detectors were 1.15 and 0.1, respectively. In other words, an image pyramid was generated while a test image was downsampled by a factor of 1.15 until the width or height of the test image gets smaller than the size of the detecting window. For one layer in the image pyramid, the detecting window slides by pixels 0.1 times of the detecting window.

**Figure 11:**
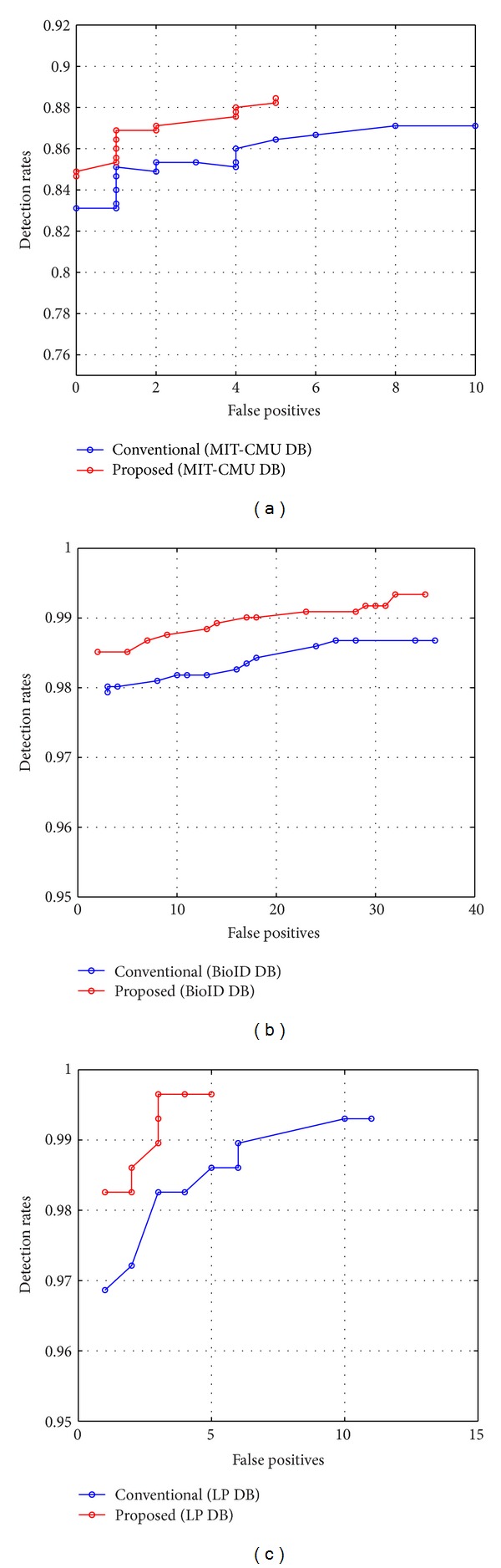
(a) Performance of face detector on MIT-CMU DB. (b) Performance of face detector on BioID DB. (c) Performance of license plate detector.

Figure 11 shows the ROCs of the face and license plate detectors for the proposed and conventional learning algorithms. The horizontal axis represents false positives representing the number of mistaken results in all tested images. The vertical axis represents detection rate calculated by the number of detected target objects divided by the total number of target objects in all the tested images. A successful detection was defined as the case when the detection result box overlaps 80% of the original target object and the area of target object exceeds half of the detection result box. To generate the ROC curve, the experiment was repeated by adjusting the sensitivity parameter *α* in the signum function for the classifier of the 5th stage of the detector as

(15) $$\begin{array}{c} H\left(\Gamma \right)=\mathrm{sign}\left(\sum_{x}p_{x}\left(\Gamma \left(x\right)\right)+\alpha \right). \end{array}$$

Figure 11(a) shows the results of the face detectors for the MIT + CMU database. Both the proposed and conventional methods produced the detection rates over 80% while maintaining less than 10 false positives, but the proposed method showed much improved performance. According to the figure, the detection rate with 5 false positives of the proposed method was 88.4% while the conventional method was 86.4%, and the detection rate with zero false positives of the proposed method was 84.9% while the conventional method was 83.1%.  Figure 11(b) shows the results of the face detectors for the BioID database. As shown in Figure 11(b), the proposed method has a higher detection rate than the conventional method with an identical number of false positives. Referring to the results, the detection rate with 30 or less false positives was 99.2% for the proposed method while it was 98.7% for the conventional method. For 10 or less false positives, the proposed method exhibited detection rate at 98.8% while the conventional method was at 98.2%. From Figure 11(c), the detection rate of the license plate detectors with 5 false positives was 99.7% for the proposed method while that of the conventional method was 98.6%.

**Figure 12:**
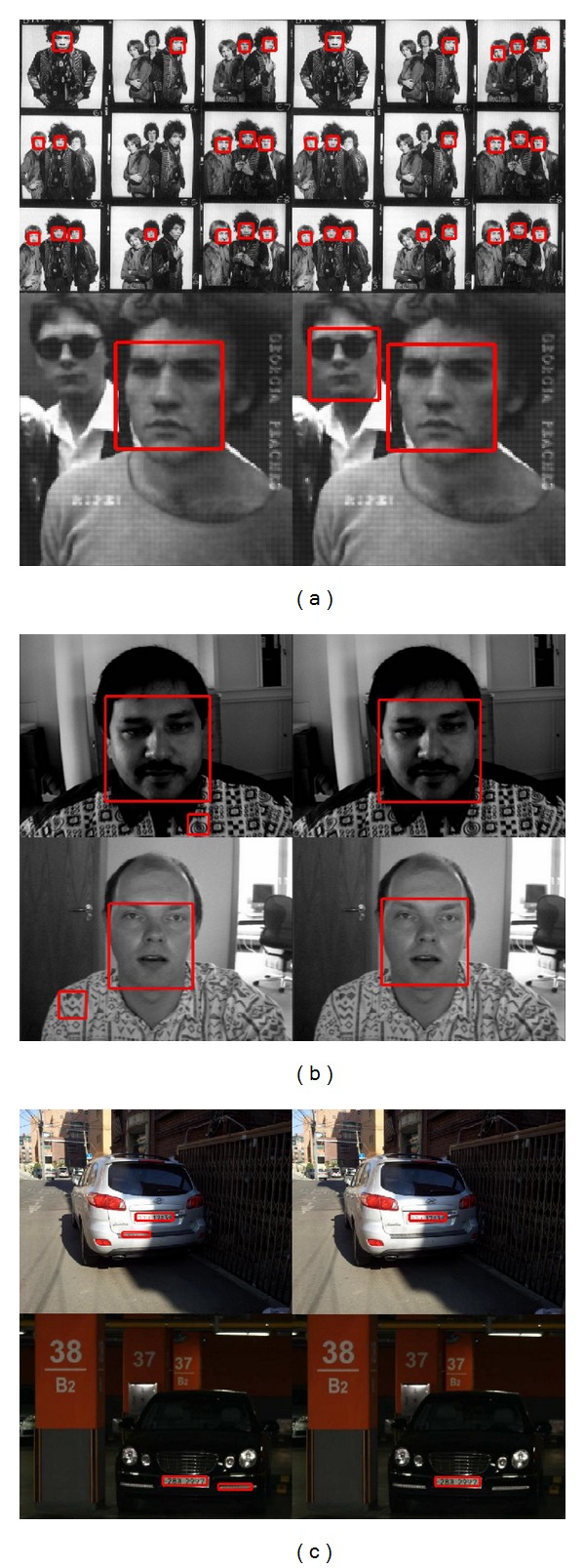
(a) Examples of detection results for MIT + CMU DB. (b) Examples of detection results for BioID DB. (c) Examples of detection results for license plate DB.

Figure 12 shows some examples of the detection results, where each of the detected target objects was framed by a box. The left half of the figure shows the results of the detector based on the conventional method, and the other half shows the results of the detector based on the proposed method. The sensitivity parameters in (15) for both detectors were set to zero. As indicated by the results, the detectors based on the proposed method have better performances meaning higher detection rates and lower false positives.

## 5. Conclusions

In this paper, we proposed a reinforced learning algorithm for image classification with LPR. The LPR based classifier was trained through a novel concept of classification implemented in a reinforced AdaBoost learning algorithm, and the AdaBoost model having an exponential criterion as a cost function was optimized by Newton's method. The novelty we claim here is that we introduced a process of selecting the weak classifier minimizing the cost function. In particular, we derived a calculation of the reinforced predictions based on confidence level in a lookup table to determine the classification results for LPR based image classifier. The proposed algorithm was able to deliver fast convergence speed by reducing the computation of combining weak classifiers for a strong classifier in the AdaBoost learning process. For performance evaluation, we measured image classification performance over face and license plate database using the MCT feature often used in the LPR based methods. Also, we have conducted an experiment to detect objects in field images with the trained object detector. From the experimental results for image classification, it was observed that the proposed learning algorithm reduced the training time by faster reduction of training error than the conventional learning algorithm while retaining higher classification performance. Results have shown that the proposed learning algorithm demonstrated better classification performance than the conventional learning algorithm. It can be stated that the proposed learning algorithm is a suitable approach to training classifiers with LPR in object detection and recognition tasks.

## Figures and Tables

**Algorithm 1:**
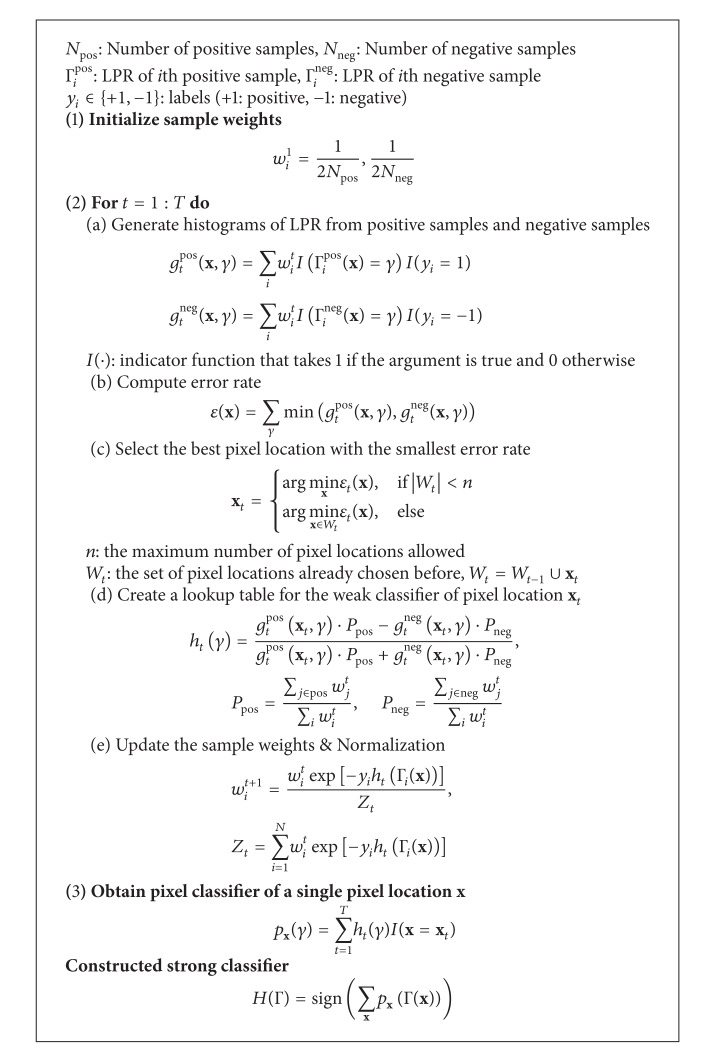
Proposed update procedure of a classifier based on a reinforced Adaboost learning.

**Table 1:** Execution time of classification and number of misclassified samples for 200,000 test samples.

Number of pixels	20	40	80	160	320
Time (sec)	0.188	0.369	0.769	1.534	3.293
Number of misclassification	101	10	3	2	0

**Table 2:** Information of the trained classifiers for face detection at each of 5 stages.

Stage		1	2	3	4	5
Rounds	Proposed	37	171	162	170	208
	Conventional	87	615	410	480	513

Pixels	Proposed	20	40	80	160	207
	Conventional	20	40	80	160	320

Dataset	Proposed	1.00 × 10^5^/1.50 × 10^5^ (1.50 × 10^5^)	1.00 × 10^5^/1.50 × 10^5^ (4.63 × 10^5^)	1.00 × 10^5^/1.50 × 10^5^ (5.71 × 10^6^)	1.00 × 10^5^/1.21 × 10^5^ (3.13 × 10^8^)	1.00 × 10^5^/0.32 × 10^5^ (3.76 × 10^9^)
	Conventional	1.00 × 10^5^/1.50 × 10^5^ (1.50 × 10^5^)	1.00 × 10^5^/1.50 × 10^5^ (4.56 × 10^5^)	1.00 × 10^5^/1.50 × 10^5^ (5.36 × 10^6^)	1.00 × 10^5^/1.29 × 10^5^ (3.13 × 10^8^)	1.00 × 10^5^/0.39 × 10^5^ (3.76 × 10^9^)

**Table 3:** Information of the trained classifiers for license plate detection at each of 5 stages.

Stage		1	2	3	4	5
Rounds	Proposed	66	254	135	137	266
	Conventional	270	1147	353	303	619

Pixels	Proposed	20	40	80	137	264
	Conventional	20	40	80	160	320

Dataset	Proposed	1.30 × 10^5^/2.16 × 10^5^ (2.16 × 10^5^)	1.30 × 10^5^/2.16 × 10^5^ (6.29 × 10^5^)	1.30 × 10^5^/2.16 × 10^5^ (4.50 × 10^7^)	1.30 × 10^5^/1.02 × 10^5^ (5.64 × 10^8^)	1.30 × 10^5^/0.78 × 10^5^ (2.32 × 10^9^)
	Conventional	1.30 × 10^5^/2.16 × 10^5^ (2.16 × 10^5^)	1.30 × 10^5^/2.16 × 10^5^ (5.99 × 10^5^)	1.30 × 10^5^/2.16 × 10^5^ (4.41 × 10^7^)	1.30 × 10^5^/1.05 × 10^5^ (5.64 × 10^8^)	1.30 × 10^5^/0.94 × 10^5^ (2.32 × 10^9^)
